# Long-Term Outcomes of Bronchopulmonary Dysplasia Under Two Different Diagnostic Criteria: A Retrospective Cohort Study at a Chinese Tertiary Center

**DOI:** 10.3389/fped.2021.648972

**Published:** 2021-03-30

**Authors:** Ling Sun, Hong Zhang, Yingying Bao, Wenying Li, Jingyuan Wu, Yuanyuan He, Jiajun Zhu

**Affiliations:** Department of Neonatology, Women's Hospital, Zhejiang University School of Medicine, Hangzhou, China

**Keywords:** bronchopulmonary dysplasia, definition, respiratory outcomes, death, pre-term infant, predictive value of tests

## Abstract

Unlike other complications among very low birth weight infants (VLBW), the incidence of bronchopulmonary dysplasia (BPD) has not decreased substantially, partly because of the different definitions of BPD applied by different researchers. In this retrospective cohort study, we aimed to compare the 2018 revised definition and the 2001 consensus definition of BPD proposed by the National Institute of Child Health and Human Development (NICHD), as well as to identify which definition better predicts severe respiratory morbidities or death. We included 417 infants born at a gestational age <32 weeks and classified them as having BPD or without BPD based on the two definitions, with a final follow-up at 18–24 months. We performed between-group comparisons of death and respiratory outcomes. Statistical analyses were performed using descriptive statistics, comparative tests, and receiver operating characteristic curves. The mean ± standard deviation gestational age and birth weight of the 417 eligible infants were 29.1 ± 1.4 weeks and 1186.6 ± 197.8 g, respectively. Among the included infants, five and three infants died before and after 36 weeks of post-menstrual age (PMA), respectively, with 68 and 344 infants evaluated at discharge and 36 weeks' PMA, respectively. We diagnosed 163 (39.1%) and 70 (16.8%) infants with BPD according to the 2001 and 2018 NICHD definitions, respectively. The 2001 NICHD definition displayed a higher sensitivity (0.60 vs. 0.28), better negative predictive value (0.89 vs. 0.85), and larger area under the receiver operating characteristic curve (0.66 vs. 0.57), but a lower specificity (0.65 vs. 0.87) and worse positive predictive value (0.26 vs. 0.31), than the 2018 definition for serious respiratory morbidity or mortality at a corrected age of 18–24 months. Compared with the 2018 NICHD definition of BPD, the 2001 NICHD consensus definition may result in more cases of false-positive or unclassified severity. However, it may be a better indicator of severe respiratory morbidities or death during the first 18–24 months. Nevertheless, there is a need for future studies to assess the validity of the new diagnostic criteria.

## Introduction

Bronchopulmonary dysplasia (BPD) is a chronic neonatal lung disease with adverse effects on pre-mature infants. It is related to adverse long-term pulmonary and neurodevelopmental outcomes, especially in very low birth weight infants (VLBW) (1–4). Over the past 50 years, unlike other complications affecting VLBW, there has been no substantial decrease in the incidence of BPD despite of improvements in neonatal care (5). The variance in the prevalence of BPD across centers has impeded between-study comparisons of BPD incidence. A major factor that affects the reported incidence rates of BPD is the definition used to classify this disease in newborns. Therefore, there is a need for accurate and timely identification of high-risk infants requiring monitoring or special support.

The definition of BPD has been debated since the first diagnostic criteria were proposed (6–12). One of the most common diagnostic criteria for BPD is proposed by the National Institute of Child Health and Human Development (NICHD) in 2001 (the 2001 NICHD definition) (6). However, recently, there have been notable changes in the perinatal and neonatal managements of pre-term infants, especially in terms of the respiratory support modalities available for pre-term neonates. The 2001 NICHD definition is limited to classify the severity of BPD, since it does not consider newer respiratory interventions (13). Consequently, in 2016, the NICHD held a workshop to revise the definition of BPD, which was reported in 2018 (the 2018 NICHD definition) (11). The 2018 definition considered newer non-invasive ventilation modes, and the severity of BPD was reclassified into grades (I, II, III, and IIIA) rather than the original terms (mild, moderate, and severe). The revision added a new category, IIIA: early death (between 14 days of postnatal age and 36 weeks) owing to persistent parenchymal lung disease and respiratory failure that cannot be attributed to other neonatal morbidities (e.g., necrotizing enterocolitis, intraventricular hemorrhage, redirection of care, episodes of sepsis, etc.). The 2018 definition was also updated to add the need for radiographic evidence of pulmonary parenchymal disease. However, the validity of the 2018 NICHD definition remains unclear.

There is a need to determine which of the available definitions more precisely predicts the long-term outcomes in discharged infants. In this study, we aimed to compare the 2001 and 2018 NICHD definitions of BPD in terms of their predictive value for serious, long-term, adverse respiratory outcomes or death.

## Materials and Methods

### Study Population and Data Collection

This retrospective study was conducted using a cohort of pre-mature infants, born at a gestational age (GA) <32 weeks, who were admitted to the neonatal intensive care unit of a tertiary hospital in Hangzhou, China, from 2016 to 2018. The inclusion criteria were as follows: admission to the neonatal intensive care unit, GA <32 weeks, birth weight <1,500 g, and survival for ≥ 14 days. The exclusion criteria were as follows: considerable congenital anomalies or death within the first 14 postnatal days, and discharge or transfer to another hospital before 34 weeks of post-menstrual age (PMA). This study was approved by the research ethics board of Women's Hospital, School of Medicine, Zhejiang University (IRB-20200213-R). Informed consent was obtained from the parents or guardian.

Maternal and infant data were collected by trained research personnel. We recorded prenatal factors, including antenatal steroid usage, maternal chorioamnionitis, small for GA (birth weight <10th percentile), preeclampsia, and maternal education. Other recorded variables included birth weight, gestational age, gender, Apgar scores at 1 and 5 min postnatally, delivery mode, surfactant administration, the duration of respiratory support and length of hospital stay. Infants were diagnosed with BPD based on both NICHD definitions.

### BPD Definitions

#### The 2001 NICHD Definition

In an NICHD workshop conducted in June 2000, a severity-based definition of BPD was proposed, in which BPD was classified as mild, moderate, or severe according to the postnatal age or PMA (6). For infants born at GA <32 weeks, BPD was defined as having required supplemental oxygen (>21%) for at least the first 28 postnatal days, and severity was evaluated at 36 weeks of PMA or at discharge, whichever occurred first. For infants born at GA ≥32 weeks, BPD was defined as having required supplemental oxygen (>21%) for at least the first 28 postnatal days, and severity was evaluated at a postnatal age of 56 days or at discharge, whichever occurred first. In terms of severity, at the time of evaluation, infants without an oxygen requirement, those requiring <30% oxygen, and those requiring positive pressure ventilation/nasal continuous positive airway pressure (PPV/NCPAP) and/or requiring ≥ 30% oxygen were diagnosed with mild, moderate, and severe BPD, respectively (6).

For this definition, an oxygen reduction test is normally used to confirm the oxygen required at a specific time point (14). However, we did not perform such tests because of the low number of eligible babies and the very high overall failure rate (70%) (10). Furthermore, the oxygen reduction test does not seem to significantly affect patient categorization, because oxygen use is more tightly used now (15).

#### The 2018 NICHD Definition

In the 2016 NICHD workshop, reported in 2018, a revised definition of BPD was proposed (11). Accordingly, BPD is graded as follows, according to respiratory support and fraction of inspired oxygen (FiO_2_) required for at least three consecutive days to maintain oxygen saturation at 0.90–0.95 in a pre-mature infant with GA <32 weeks, who presents with persistent, radiographically confirmed parenchymal lung disease at 36 weeks of PMA.

Grade I: NCPAP/non-invasive intermittent PPV/nasal cannula with a flow rate of ≥ 3 L/min and an FiO_2_ of 0.21; a nasal cannula with a flow rate of 1–3 L/min, or a hood and an FiO_2_ of 0.22–0.29; or a nasal cannula with a flow rate of <1 L/min and an FiO_2_ of 0.22–0.70.

Grade II: Invasive intermittent PPV with an FiO_2_ of 0.21; NCPAP/non-invasive intermittent PPV/nasal cannula with a flow rate of ≥ 3 L/min and an FiO_2_ of 0.22–0.29; a nasal cannula with a flow rate of 1–3 L/min, or a hood and an FiO_2_ ≥ 0.30; or a nasal cannula with a flow rate of <1 L/min and an FiO_2_ of > 0.70.

Grade III: Invasive intermittent PPV with an FiO_2_ > 0.21; or NCPAP/non-invasive intermittent PPV/nasal cannula with a flow rate of ≥ 3 L/min with an FiO_2_ ≥ 0.30.

Grade IIIA: Early death (from 14 days after birth to 36 weeks of corrected GA) caused by persistent parenchymal lung disease and respiratory failure, with the exclusion of death caused by necrotizing enterocolitis, severe intraventricular hemorrhage, sepsis, and other neonatal conditions (11).

### Outcomes

The composite primary outcome was mortality or severe respiratory morbidity from 36 weeks of PMA to the corrected 18–24 month follow-up. Severe respiratory morbidity was defined as any of the following (16–20): having undergone a tracheostomy; hospitalized for respiratory reasons at ≥ 50 weeks of PMA; requiring supplemental oxygen, respiratory support, or respiratory surveillance (e.g., pulse oximeter or apnea monitor) at the 18–24 month follow-up; and having ≥ 2 re-hospitalizations for respiratory reasons before the 18–24 month follow-up. In this cohort, ≥ 2 re-hospitalizations represented the 75th percentile of the number of re-hospitalizations.

During the follow-up period, which ended in August 2020, questionnaires were administered to the parents/caregivers to obtain post-discharge respiratory outcome data. The questionnaire contained items regarding respiratory support (including tracheostomy and mechanical ventilation), requirement for supplemental oxygen or respiratory surveillance (e.g., pulse oximeter or apnea monitor), history of respiratory symptoms (cough without having a cold/wheeze at least once per week), airway-infection frequency, and hospital readmissions for respiratory reasons. We excluded participants whose parents/caregivers did not answer questions or could not be contacted. We also recorded hospitalizations for respiratory indications, respiratory support (including tracheostomy and mechanical ventilation), requirement of supplemental oxygen or respiratory surveillance, and late death.

### Statistical Analysis

For descriptive statistics, continuous variables were presented as means ± standard deviations or medians with interquartile ranges, while categorical variables were presented as frequencies and percentages. Chi-square test and McNemar's-test was used for between-definition comparisons of incidence rates. Further, we established receiver operating characteristic curves to identify the best definition for outcome prediction. Moreover, we calculated the sensitivity, specificity, and the positive and negative predictive values of the BPD definitions for predicting long-term outcomes. All statistical analyses were performed using IBM SPSS Statistics for Windows, version 26.0 (IBM Corp., Armonk, NY, USA). All statistical tests were two-sided with statistical significance set at *P* < 0.05.

## Results

**Figure 1:**
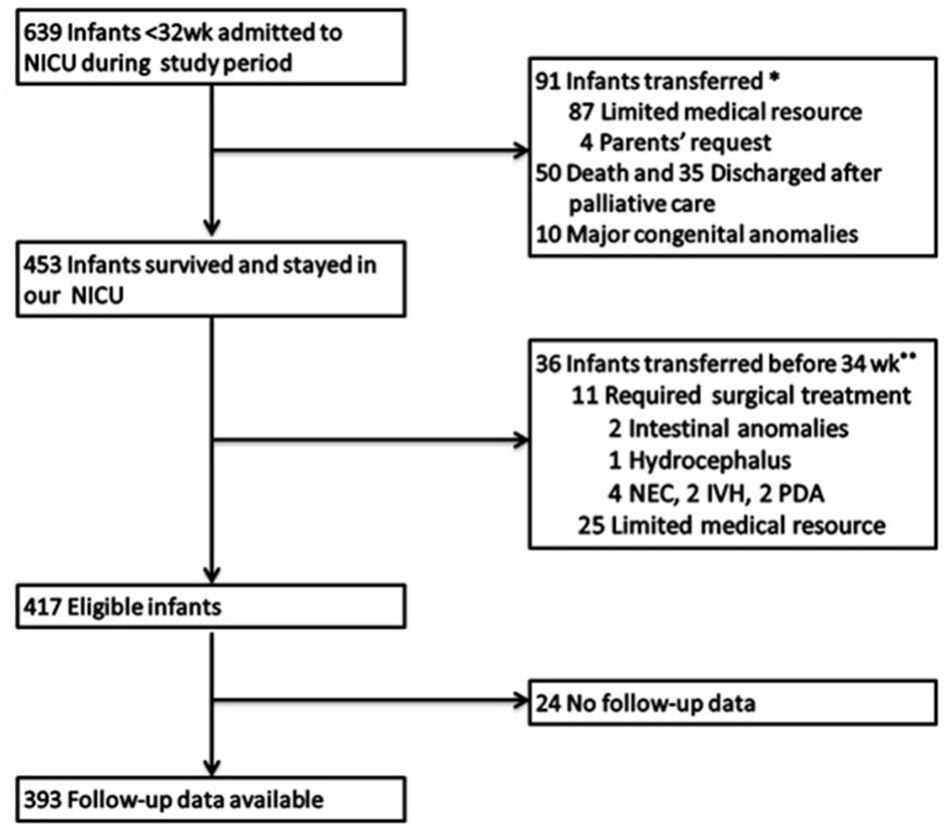
Study population. NICU, neonatal intensive care unit; NEC, necrotizing enterocolitis; IVH, severe intraventricular hemorrhage; PDA, patent ductus arteriosus. ^*^Early transfer: The 91 patients transferred early just after birth. Among them 87 patients transferred to another tertiary center in our city due to limited medical resources and 4 patients were transferred to other hospitals for parents' request. ^**^Late transfer: The 36 patients transferred before 34 weeks PMA included two parts: 25 were due to limited medical resources and 11 required surgical treatment, for the lack of surgery department in our hospital.

Among 639 infants (GA <32 weeks) who were admitted to our neonatal intensive care unit during the study period, 417 met the inclusion criteria (Figure 1). Among them, 5 (1.2%) infants died before 36 weeks of PMA, with 68 and 344 infants evaluated at discharge and 36 weeks of PMA, respectively. Further, 24 (5.8%) infants were lost to follow-up and were excluded from the analysis of long-term outcomes.

Table 1 presents the maternal and infant characteristics at birth. The mean GA and birth weight were 29.1 ± 1.4 weeks and 1186.6 ± 197.8 g, respectively; 53.7% of infants were male. Additionally, 348 (83.5%) infants received oxygen and 11.8% were intubated in the delivery room. More than two thirds of infants were merely receiving non-invasive respiratory support during the hospitalization. Additionally, we performed surfactant administration mostly by the INSURE method. About 56.6% of the infants received a surfactant dose and 1.9% received more than one surfactant dose. The average length of hospital stay was 61.1 ± 21.7 days.

**Table 1:** Demographic characteristics of the study population (*n* = 417).

**Characteristic**	**Value**
Gestational age, weeks, mean (SD)	29.1 (1.4)
Birth weight, g, mean (SD)	1186.6 (197.8)
Male, *n* (%)	224 (53.7)
Female, *n* (%)	193 (46.3)
Cesarean section, *n* (%)	261 (62.6)
Multiple birth, *n* (%)	146 (35.0)
SGA, *n* (%)	23 (5.5)
IUGR, *n* (%)	20 (4.8)
Apgar score 1 min, median (IQR)	9 (7, 9)
Apgar score 5 min, median (IQR)	10 (9, 10)
Chorioamnionitis, *n* (%)	61 (14.6)
GDM, *n* (%)	60 (14.4)
Hypertensive disorders of pregnancy, *n* (%)	100 (24.0)
Any antenatal steroids, *n* (%)	382 (91.6)
At least 1 complete course, *n* (%)	298 (71.5)
Maternal education < high school, *n* (%)	121 (29.0)
Intubation, *n* (%)	49 (11.8)
1st dose surfactant, *n* (%)	236 (56.6)
2nd dose surfactant, *n* (%)	8 (1.9)
Mechanical ventilation, *n* (%)	87 (20.9)
Non-invasive respiratory support, *n* (%)	284 (68.1)
Gestational age at discharge, weeks, mean (SD)	37.9 (2.3)
Length of stay, d, mean (SD)	61.1 (21.7)
Respiratory support, d, median (IQR)	20.0 (6.0, 41.0)
Oxygen use, d, median (IQR)	1.0 (0.0, 19.7)

*SD, standard deviation; IUGR, intrauterine growth retardation; SGA, small for gestational age; IQR, interquartile range; GDM, gestational diabetes mellitus*.

Regarding BPD classification, 163 (39.1%) and 70 (16.8%) infants were classified as having BPD using the 2001 and 2018 NICHD definitions, respectively. Thus, 57.1% of the infants diagnosed with BPD based on the 2001 definition were negative according to the 2018 definition. Table 2 presents the classification of the infants based on the two definitions. Using the 2001 NICHD definition, 88 (21.1%), 44 (10.5%), and 14 (3.4%) infants were diagnosed with mild, moderate, and severe BPD, respectively. Furthermore, the BPD severity of 17(4.1%) infants could not be classified using the 2001 definition. Using the 2018 definition, 48 (11.5%), 8 (1.9%), 9 (2.2%), and 5 (1.2%) infants were diagnosed with grades I, II, III, and IIIA BPD, respectively. There was a significant between-definition difference in the BPD incidence (chi-square-test *P* < 0.001 and McNemar's-test *P* < 0.001).

**Table 2:** Number of infants with BPD according to the 2001 and 2018 NICHD definitions.

	**Mild/I**	**Moderate/II**	**Severe/III**	**IIIA**	**Unclassified**	**BPD**	**No BPD**
2001 BPD	88 (21.1)	44 (10.5)	14 (3.4)	–	17 (4.1)	163 (39.1)	254 (60.9)
2018 BPD	48 (11.5)	8 (1.9)	9 (2.2)	5 (1.2)	0 (0.0)	70 (16.8)	347 (83.2)

*Values are presented as numbers (percentages)*.

*BPD, bronchopulmonary dysplasia; NICHD, National Institute of Child Health and Human Development*.

Sixty-seven (17.2%) infants had an outcome of respiratory morbidity and/or late death after discharge at a corrected age of 18–24 months; among them, 3 (0.7%) infants died after initial discharge, while 64 (16.5%) experienced serious respiratory morbidity. Table 3 presents the rates of late death or serious respiratory morbidity stratified according to infants with and without BPD, classified according to both the diagnostic criteria.

**Table 3:** Late death or serious respiratory morbidity rates for different BPD severities. *(n* = 388)^*^.

	2001 BPD (***n***=152)^*^					2018 BPD (***n***=62)^*^			
	**No**	**Mild**	**Moderate**	**Severe**	**Unclassified**	**No**	**I**	**II**	**III**
Death after 36 weeks' PMA	1 (0.4)	1 (0.7)	0 (0.0)	0 (0.0)	1 (0.7)	2 (0.6)	1 (1.6)	0 (0.0)	0 (0.0)
Tracheostomy	1 (0.4)	0 (0)	0 (0.0)	0 (0.0)	0 (0.0)	1 (0.3)	0 (0.0)	0 (0.0)	0 (0.0)
NICU hospitalization beyond 50 weeks' PMA for respiratory reasons	0 (0.0)	0 (0.0)	1 (0.7)	2 (1.3)	0 (0.0)	0 (0.0)	1 (1.6)	0 (0.0)	2 (3.2)
Supplemental O_2_ use at follow-up	1 (0.4)	0 (0.0)	0 (0.0)	2 (1.3)	1 (0.7)	1 (0.3)	0 (0.0)	1 (1.6)	2 (3.2)
Ventilator or NCPAP at follow-up	1 (0.4)	0 (0.0)	2 (1.3)	0 (0.0)	0 (0.0)	1 (0.3)	2 (3.2)	0 (0.0)	0 (0.0)
Respiratory monitor use at follow-up	1 (0.4)	0 (0.0)	0 (0.0)	2 (1.3)	1 (0.7)	1 (0.3)	0 (0.0)	1 (1.6)	2 (3.2)
≥2 hospitalizations for respiratory reasons	27 (11.4)	14 (9.2)	8 (5.3)	3 (2.0)	9 (5.9)	48 (14.7)	7 (11.3)	3 (4.8)	3 (4.8)
Total^**^	27 (11.4)	14 (9.2)	11 (7.2)	4 (2.6)	11 (7.2)	48 (14.7)	10 (16.1)	5 (8.1)	4 (6.4)

* *Data are shown for patients who underwent follow-up at 18–24 months' corrected age. Patients of grade IIIA BPD were not included*.

** *Data are shown for the number of patients*.

*Values are presented as numbers (percentages)*.

*BPD, bronchopulmonary dysplasia; PMA, post-menstrual age; NICU, neonatal intensive care unit; NCPAP, nasal continuous positive airway pressure*.

**Figure 2:**
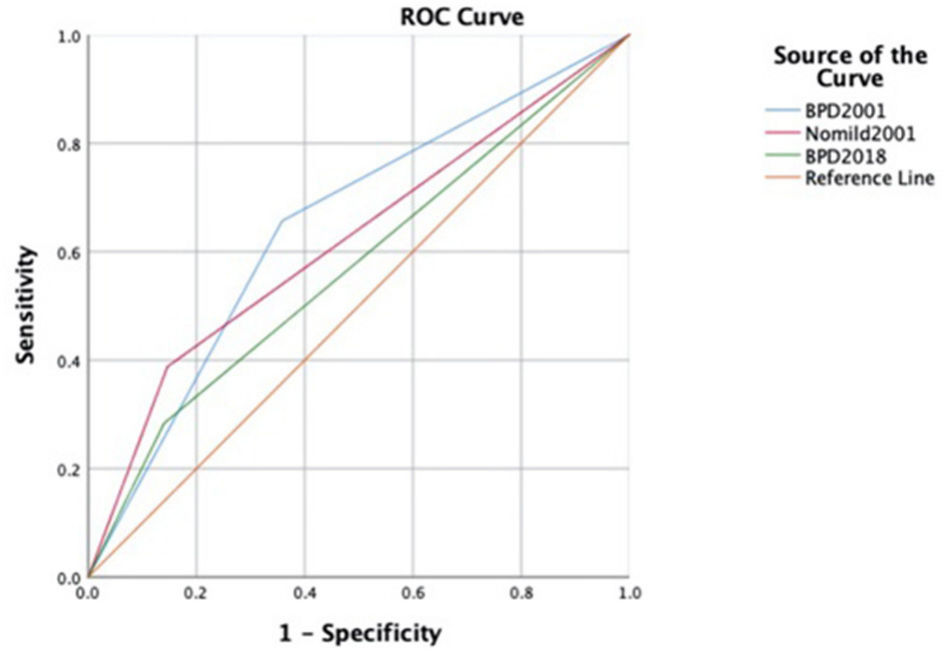
ROC curves to predict late death or serious respiratory morbidity in different definitions. ROC, receiver operating characteristic curve.

The areas under the receiver operating characteristic curves (AUCs) revealed that the 2001 NICHD definition of BPD had a higher sensitivity, better negative predictive value, and larger AUC on mortality or serious respiratory morbidity than the 2018 definition at a corrected age of 18–24 months (AUC: 0.66 vs. 0.57). When the “mild” categories of BPD were removed from the 2001 definition, its specificity increased (from 0.65 to 0.86), but its sensitivity decreased (from 0.60 to 0.39), and its AUC decreased (from 0.66 to 0.62) (Figure 2).

## Discussion

In this retrospective cohort study, we compared the predictive values of the 2001 and 2018 NICHD definitions of BPD with respect to serious respiratory morbidities and late death. Notably, at a corrected age of 18–24 months, the 2001 NICHD definition was a better predictor of late death or serious respiratory morbidity than the 2018 definition.

In reports published between 1978 and 2015, the incidence of BPD has ranged from 6 to 59% based on different definitions (10, 21). In 2005, Ehrenkranz et al. reported BPD incidence rates of 44 and 77% when diagnosed according to oxygen use at 36 weeks of PMA and the 2001 NICHD definition, respectively (16). In our study, the 2001 definition yielded a significantly higher BPD incidence than the 2018 definition (*P* < 0.001). It may be caused mostly by different definitions, because the 2001 NICHD definition put more attention on the course of oxygen requirement, while the situation of VLBW at 36 weeks of PMA was more powerful in the 2018 definition. Fewer infants met the criterion of requiring supplemental oxygen at 36 weeks of PMA (a requirement in the 2018 NICHD definition) than those who met the criterion of requiring supplemental oxygen for the first 28 postnatal days (the only absolute requirement in the 2001 NICHD definition) (16). Furthermore, we also observed a relatively low proportion of infants with severe BPD (or grade III), which was inconsistent with the findings of Wang et al.'s study (22), which reported a high proportion of infants with severe BPD. This may be attributed to the mainstream of participants, who were well-managed prenatally and postnatally in the same unit. We had implemented a unified and standardized management protocol, especially in terms of respiratory support and oxygen use in our unit. We speculated that the custom of respiratory support and the source of VLBW should be considered, when we applied the BPD definition.

Given the advancements in new modes of non-invasive respiratory support, there has been an increase in the proportion of BPD cases for which the severity could not be classified under the 2001 definition. In terms of those requiring high flow nasal cannula at 36 weeks of PMA after oxygen treatment for at least 28 days, the patients met the 2001 definition of BPD, but they could not be classified by the 2001 definition. In our study, the severity of BPD of 17 infants (4.1%) could not be classified using the 2001 NICHD definition, which was consistent with the reported proportion of 2–16% in the study by the Pre-maturity and Respiratory Outcomes Program, using three different definitions (10).

Our main finding that 2001 NICHD definition could more accurately predict adverse pulmonary outcomes during the first 18–24 months, was consistent with several previous studies. Ehrenkranz et al. (16) discovered that the 2001 NICHD definition could more accurately predict adverse pulmonary and neurodevelopmental outcomes than the other definitions, including those with the only criterion being the requirement of supplemental oxygen for the first 28 postnatal days or at 36 weeks of PMA. Davis et al. (20) reported that the duration of oxygen therapy was a weak predictor of long term pulmonary abnormalities. Smith et al. (23) also reported a better predict value between the BPD and an increased re-hospitalization rate. However, our finding was inconsistent with the following reported studies. In 1988, Shennan et al. (24) reported that the requirement of supplemental oxygen at 36 weeks of PMA, rather than for the 28 consecutive days of oxygen supplement, was the better predictor of adverse pulmonary function (with about 63% sensitivity and 91% specificity), during the first 2 years of life. Jensen et al. (12) reported that a definition similar with the 2001 NICHD definition was a less accurate predictor of long term outcomes than a definition similar with the 2018 NICHD definition. These inconsistencies may be attributed to differences in the different characteristics of study population and the indices used (accuracy vs. c-statistics or AUCs). Concerned with the different options on mild BPD and the overestimated cases under 2001 definition, we also conducted a new AUC curve by combining no BPD and mild BPD as a total. We still found that the 2001 NICHD definition was relatively more powerful than the 2018 definition in the issue of adverse respiratory status during the first 18-24 months. It was speculated that the course of respiratory support, including oxygen supplements rather than the respiratory condition at a certain time (36 weeks of PMA) should be considered more, when we considered the long term respiratory morbidities in VLBW.

Our study had several strengths. Firstly, and most importantly, all infants included in this study were born and managed in the same unit and underwent a unified and standardized management with the consistent indications for respiratory support, which adds to the plausibility of the difference in outcomes. Secondly, this study had a high follow-up rate; moreover, the follow-up assessment was relatively simple and objective, which decreased the bias of our findings. Thirdly, we determined the sensitivity and specificity, as well as the positive and negative predictive values of the different BPD definitions, which confirmed the robustness of our findings. To the best of our knowledge, this is the only study, which was conducted in one unit, to assess the difference in BPD incidence using the two NICHD definitions, as well as to report the predictive values for death and/or serious respiratory morbidity at a corrected age of 18–24 months. These findings provide a theoretical basis for establishing new diagnostic criteria.

Our study also had several limitations. Firstly, it was a single center study without large number of infants. More standard diagnosis, as well as unified prenatal and postnatal managements may reduce some variable factors. Secondly, there were a relatively high proportion of infants, who were excluded from analyses due to transfer. There was a possibility of selection bias, because a small number of patients transferred owing to necrotizing enterocolitis, severe intraventricular hemorrhage, patent ductus arteriosus (listed in Figure 1). However, the main cause of transfer was the limited medical resources, and there were no significant differences between the characteristics of the transferred and included populations. Thirdly, the comparison of the combined outcomes of death, adverse respiratory status and neurological outcome during the first 18–24 months between different definition, may give us more information, however, neurodevelopmental assessment may be affected by various factors, such as different assessment tools and personal experience. Jensen et al. (12) and Isayama et al. (19) had the similar opinion on the very weak relationship between the predicted value of BPD definitions and neurodevelopmental impairment. Further study may focus on this issue. Finally, in our study, we chose to compare the two NICHD definitions. The optimal diagnosis of BPD by Jensen et al. (12) was not included to compare, because we found the characteristics of the mainstream population in our study were different to those in Jensen's study. The infants with mean gestational age of 29 weeks of gestation, was not the population at the highest risk of BPD. We speculated that the issue of characteristics of infants should be also considered, when further relevant studies were conducted.

In conclusion, the 2001 NICHD definition of BPD yielded a higher sensitivity, better negative predictive value, and larger AUC for death or serious respiratory morbidity at a corrected age of 18–24 months, when compared with the 2018 definition in VLBW. To establish an improved BPD definition, the limitations of the current definitions should be clearly defined. There is a need for future studies to assess the validity of the new diagnostic criterion. Moreover, there is a need to establish a more comprehensive BPD definition based on early pathological and biomarker identification.

## Data Availability Statement

The original contributions generated for the study are included in the article/Supplementary Material, further inquiries can be directed to the corresponding author.

## Author Contributions

LS had primary responsibility for protocol development, outcome assessment, preliminary data analysis, and writing of the manuscript. HZ and WL participated in the design of the protocol, patient screening, and enrollment. YB participated in the protocol design and was responsible for all statistical analyses. JW and YH contributed to patient screening and writing of the manuscript. JZ supervised the design and execution of the study, performed the final data analyses, and contributed to the writing of the manuscript. All authors contributed to the article and approved the submitted version.

## Conflict of Interest

The authors declare that the research was conducted in the absence of any commercial or financial relationships that could be construed as a potential conflict of interest.
