# Supplementary material for: Long-Term Outcomes of Bronchopulmonary Dysplasia Under Two Different Diagnostic Criteria: A Retrospective Cohort Study at a Chinese Tertiary Center
Source: Front Pediatr. 2021 Mar 30;9:648972. doi: 10.3389/fped.2021.648972 (PMC8042161; doi:10.3389/fped.2021.648972)
Supplement: Supplementary file 1 [file Table_1.pdf]

Table 1. Clinical characteristics of the included and excluded population.

Characteristic	Included (n=417)	Excluded (n=222)	P value
Gestational age, weeks, mean (SD)	29.1 (1.4)	28.9 (1.5)	.10
Birth weight, g, mean (SD)	1186.6 (197.8)	1167.9 (199.3)	.26
Male, n (%)	224 (53.7)	116 (52.3)	.20
Cesarean section, n (%)	261 (62.6)	140 (63.1)	.13
SGA, n (%)	23 (5.5)	12 (5.4)	.008*
Apgar score 1 min, median (IQR)	9 (7, 9)	9（6， 9）	.55
Apgar score 5 min, median (IQR)	10 (9, 10)	9（9， 10）	.88
Chorioamnionitis, n (%)	61 (14.6)	29 (13.1)	.11
GDM, n (%)	60 (14.4)	34 (15.3)	.09
Hypertensive disorders of pregnancy, n (%)	100 (24.0)	51 (23.0)	.12
Antenatal steroids, n (%)	382 (91.6)	110 (49.5)	<.001*
Maternal education < high school, n (%)	121 (29.0)	72 (32.4)	.34

*Differences significant. All other differences not significant.

SD, standard deviation; SGA, small for gestational age; IQR, interquartile range; GDM, gestational diabetes mellitus
